# Can diffuse reflectance spectroscopy identify shuntodynia in pediatric hydrocephalus patients?

**DOI:** 10.1117/1.JBO.29.3.037002

**Published:** 2024-03-12

**Authors:** Olivia Kline, Karthik Vishwanath, Boyd Colbrunn, Andrew Peachman, Jing Zhang, Sudhakar Vadivelu

**Affiliations:** a Miami University, Department of Physics, Oxford, Ohio, United States; b Miami University, Department of Statistics, Oxford, Ohio, United States; c Cincinnati Children’s Hospital Medical Center, Department of Neurosurgery, Cincinnati, Ohio, United States

**Keywords:** vascular nociception, tissue optics, *in vivo*, visible-near infrared, optical spectroscopy

## Abstract

**Significance:**

Shuntodynia is patient reported pain at the site of the implanted ventriculoperitoneal (VP) shunt. Pediatric hydrocephalus requiring shunt placement is a chronic and prevalent standard of care treatment and requires lifetime management. Shuntodynia is a subjective measure of shunt dysfunction. Quantitative, white-light tissue spectroscopy could be used to objectively identify this condition in the clinic.

**Aim:**

Pediatric subjects were recruited for optical sensing during routine clinical follow-up visits, post-VP shunt implantations. Acquired optical signals were translated into skin-hemodynamic signatures and were compared between subjects that reported shuntodynia versus those that did not.

**Approach:**

Diffuse reflectance spectroscopy (DRS) measurements were collected between 450 and 700 nm using a single-channel fiber-optical probe from (N=35) patients. Multiple reflectance spectra were obtained by the attending physician from regions both proximal and distal to the VP shunt sites and from a matched contralateral site for each subject. Acquired reflectance spectra were processed quantitatively into functional tissue optical endpoints. A two-way, repeated measures analysis of variance was used to assess whether and which of the optical variables were statistically separable, across subjects with shuntodynia versus those without.

**Results:**

Analyses indicated that intrapatient differences in vascular oxygen saturation measured between shunt sites relative to that obtained at the scar or contralateral sites was significantly lower in the pain group. We also find that the total hemoglobin concentrations at the shunt site were lowest relative to the other sites for subjects reporting pain. These findings suggest that shuntodynia pain arises in the scalp tissue around the implanted shunts and may be caused due to hypoxia and inflammation.

**Conclusions:**

Optically derived hemodynamic variables were statistically significantly different in subjects presenting with shuntodynia relative to those without. DRS could provide a viable mode in routine bedside monitoring of subjects with VP shunts for clinical management and assessment of shuntodynia.

## 1 Introduction

Hydrocephalus is characterized by an increased intracranial cerebrospinal fluid volume that is independent of hydrostatic or barometric pressure.^1^ This condition is prevalent and costly with some sources estimating that hydrocephalus costs the United States healthcare system nearly two billion dollars annually.^2^^,^^3^ Hydrocephalus can be a result of several ailments including tumors, degenerative diseases, or trauma and may occur at any age and is also known to arise congenitally in pediatric patients (with one case of hydrocephalus for every 1000 births).^1^^,^^4^ With a diverse range of associated symptoms, hydrocephalus reduces the quality of life for child-patients during diagnostic evaluation and possibly even after treatment.

Ventriculoperitoneal (VP) shunts are the gold standard in treating pediatric hydrocephalus.^5^ VP shunts, however, elicit challenges for patients including infection, mechanical, and functional issues.^6^ Additionally, nearly half of pediatric hydrocephalus patients reported headaches and generalized pain after VP shunt placements.^7^ Here we use the term shuntodynia to indicate patient-reported pain that is reported clinically in patients with an implanted VP shunt. Specifically, this pain can occur at the shunt valve site or more distally along the shunt distal tubing. Interestingly, shuntodynia has been reported with and without shunt malfunctions.^8^^,^^9^ Though clinically recognized, the condition has not received significant attention since it is usually seldom life-threatening. The presence of shuntodynia is indicative of patient discomfort, and there is significant physical and psychosocial burden associated with implanted shunt devices.^10^

Optical sensing techniques, such as near infrared spectroscopy (NIRS), are well suited to explore questions pertaining to pain since they are noninvasive, painless and can be acquired during noxious stimuli.^11^ The use of such methods for objective assessment of pain has widely been explored through measurement of hemodynamic responses in various brain regions via functional NIRS.^12^^–^^15^ Optical spectroscopic methods have also been widely used to monitor and assess pain in exercise science and sports medicine.^16^^–^^19^ Parallelly, a related diffuse optical technique called diffusion correlation spectroscopy has been used in pediatric subjects with hydrocephalus for rapid and noninvasive assessment of intracranial pressure.^20^^–^^24^ However, to the best of our knowledge optical sensing has not been applied or explored in the realm of shuntodynia thus far. Additionally, most previous studies that have used optical sensing for pain studies have mostly focused on using these methods for detecting functional changes (such as blood oxygenation, volume, or tissue perfusion) from deep tissue layers. Again, to the best of our knowledge no previous diffuse optical studies have examined optically derived functional changes in skin tissues.

We have previously employed fiber-based diffuse reflectance spectroscopy (DRS) systems operating at short source–detector geometries to quantify functional properties in skin tissues *in vivo*.^25^^–^^27^ We have also previously reported preliminary findings from our use of DRS in pediatric patients implanted with VP shunts for hydrocephalus with or without shuntodynia.^28^ Here we extend our analysis to be statistically robust and examine if patient reported shuntodynia was detectable using optically derived, quantitative hemodynamic signatures obtained in skin-tissues adjacent to VP implants in subjects during a single clinical visit. In our investigation, we hypothesize that shuntodynia-related pain signals originate in or around the scalp-skin sensory system that reside above the skull where the shunt is implanted. Since this region is quite superficial (top 0.3 to 0.5 mm of the skin surface), we use optical probes with shallow-depth sensitivity.

## 2 Methods

### 2.1 Study Protocol and Clinical Data collection

All procedures for data collection were conducted with approval from the Institutional Review Board at Cincinnati Children’s Hospital Medical Center. Informed consent was obtained from the patient’s parent or legal guardian during routine clinic appointments. Invitation to participate in the study was offered to pediatric hydrocephalus patients that had a VP shunt at the discretion of the attending neurosurgeon. Patients with an inability to stay still for duration of DRS scans (which ranged between 5 and 15 min across subjects) or those that presented hypersensitivity to placement of optical probe were excluded. Of the 35 patients (N=9 with shuntodynia) that were recruited in the study, data from four subjects were missing (due to computer glitches), and one subject had VP shunts on both sides and was excluded. Thirty subjects (N=8 with shuntodynia) had complete datasets and were used for the analysis reported here. 16 subjects were female and the age range of subjects recruited for these studies ranged between 3 and 20 years. Skin tones ranged from white Caucasian–African–American and Fitzpatrick scores were not collected. However, in data analysis of the reflectance (as described below), melanin was used as an independent absorber by the inverse model to mitigate impact of skin tone on recovered hemoglobin variables. Subjects’ pain thresholds were assessed using the subjective but widely used Faces Pain Scale.^29^

**Fig. 1:**
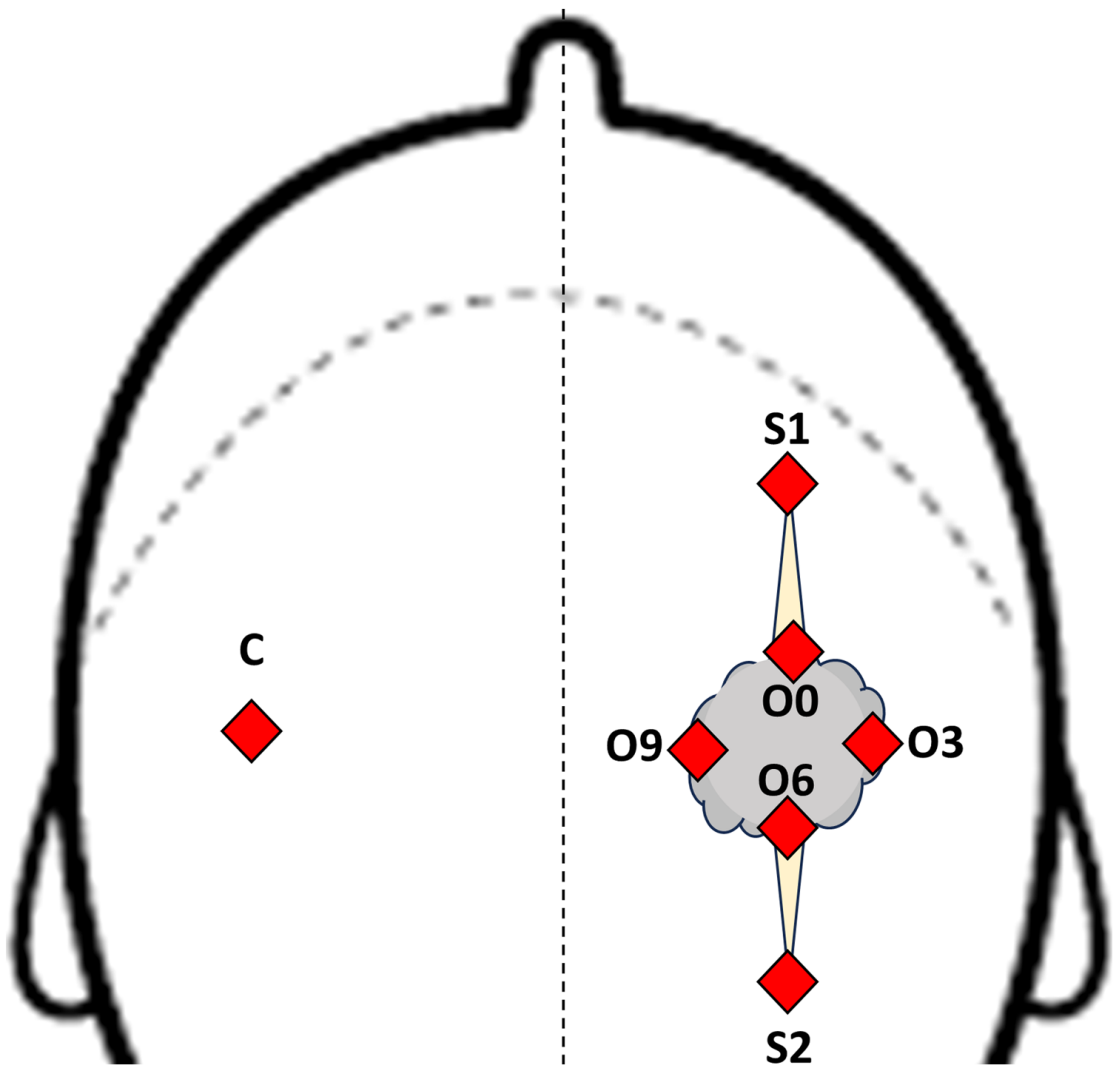
Schematic depiction of the anatomical placement of the optical probes from a top view of the head. Placement of probes are shown as filled diamonds for the seven sites. Shunt sites are O0-O9 (represent O’clock positions) were approximately distributed around a circle the size of a US quarter (2.5 cm), with center atop the shunt. Sites S1 and S2 represent scar sites (S1 and S2 were located at edge of surgical scars) and were 3 to 5 cm away from closest O’clock sites (O0 and O6, respectively). C is the contralateral matched location on the opposite side of the head relative to the shunt. The diameter of the fiber optical probe used was lesser than 1.5 mm.

Optical data collection was performed by having the patients in the seated position. DRS scans were collected at seven different spatial sites for N=25 subjects (four sites circling the shunt labeled by O’clock positions, two at the scar sites proximal and distal to shunt, and one from a matched contralateral site on the opposite side to where the shunt was implanted) as a within-subjects control. The placement of the optical probes is indicated schematically by Fig. 1. For five subjects, data from the contralateral sites were not stored correctly and thus not available.

### 2.2 Instrumentation

DRS measurements were obtained using a fiber spectroscopy system described previously.^25^^,^^28^ The system was constructed by coupling a broadband tungsten–halogen light source (HL2000, Ocean Optics, Florida, United States) to the tissue using a bifurcated optical fiber with core size 600  μm and collecting the diffuse reflectance at a distance of ∼600  μm from the source, with an identical fiber (BIF600-UV-VIS, Ocean Optics, Florida, United States). The average depth would not exceed 300 to 500  μm with these probe dimensions and optical spectrum and thus would be ideal to probe vascular responses in superficial layers of the scalp-skull tissue layers.

The collected diffuse reflectance was directed to the entrance slit of a spectrometer (USB4000, Ocean Optics, Florida, United States) for collection. On each day, prior to data collection from the patient, baseline reference spectra were collected from a white spectralon reflectance standard (Labsphere, North Sutton, New Hampshire, United States) and from a homogeneous solid reference phantom with known optical properties (INO, Hamilton, Ontario, Canada) for calibration and are summarized below. Integration times on the spectrometer for collection of a single DRS scan collection ranged between 10 and 35 ms. For each subject, dark scans were also obtained with the probe placed on subjects but with lamp shuttered. For each tissue site, five consecutive scans were collected and stored.

### 2.3 Data Fitting and Filtering

Optical measurements acquired from subjects were analyzed using MATLAB (Mathworks, Natick, Massachusetts, United States). Each spectrum was corrected for spectrometer integration time, dark-noise subtracted, and then divided (wavelength–wavelength) by the reflectance spectrum obtained from a white spectralon reflectance standard, to obtain a DRS spectrum that could be inverse-fitted using a previously reported scalable Monte Carlo (MC)-based photon transport model in a semi-infinite medium that has been well described and widely used.^30^

The scalable MC model models the source–detector geometry used experimentally and rapidly computes the diffuse reflectance spectrum from a semi-infinite homogenous medium, when given inputs of the absorption and scattering spectra, $\mu_{a}(\lambda )$ and $\mu_{s}(\lambda )$. In the inverse mode, these inputs are constructed by optimizing for inputs $c_{i},A$, and b, where $\mu_{a}(\lambda )=\overset{N}{\underset{i=i}{\sum }}(c_{i}\cdot \varepsilon_{i}(\lambda ))$ and $\mu_{s}^{\prime }(\lambda )=A\cdot \lambda^{-b}$. It is important to note that obtaining optical absorption is only possible with knowledge of the extinction coefficients $\varepsilon_{i}(\lambda )$ for each of the N absorbers that must be assumed present in the medium measured. For our modeling measurements obtained here, it was assumed that there were N=4 absorbers (oxy-hemoglobin, deoxy-hemoglobin, eumelanin, and pheomelanin^31^) and the model optimized for a total of six parameters.^32^ Each measured DRS was fit by the model where the fit was optimized by adjusting these six parameters so that modeled data fit measured spectra. For the inverse analyses, each reflectance spectra was linearly interpolated between 450 and 700 nm to provide 125 spectral points that were used to optimize the six parameters. The inverse MC was implemented using the nonlinear least squares optimizer in MATLAB (Mathworks, Natick, Massachusetts, United States).^26^^,^^27^

At convergence, the absorption coefficient directly gave oxygenated and deoxygenated hemoglobin molar concentrations ($c_{1}=[{\mathrm{HbO}}_{2}]$ and $c_{2}=[\mathrm{dHb}]$), which were added to yield the total hemoglobin concentration ([THb]). Vascular oxygen saturation was calculated as a ratio of oxygenated hemoglobin to total hemoglobin and reported as a percent value where ${\mathrm{SO}}_{2}=100\times [{\mathrm{HbO}}_{2}]/[\mathrm{THb}]$. The scattering coefficient $\mu_{s}(\lambda )$ was averaged across the spectrum to give a mean value $<\mu_{s}>$. Thus each DRS measurement after inverse fitting provided three scalar values of ${\mathrm{SO}}_{2}$, [THb] and $<\mu_{s}>$.

For each DRS spectrum fitted by the inverse model, a goodness of ($\chi^{2}$) was calculated as

$$\chi^{2}=1-\frac{\sum {\left(R_{m}(\lambda_{j})-R_{f}(\lambda_{j})\right)}^{2}}{\sum {\left(R_{m}(\lambda_{j})-{\overset{´}{R}}_{m}\right)}^{2}}.$$ (1)

In Eq. (1), $R_{m}(\lambda_{j})$ and $R_{f}(\lambda_{j})$ are measured and fitted DRS, respectively, with the sum computed across all 125 wavelengths (indexed by j). ${\overset{´}{R}}_{m}$ was the wavelength-averaged measured reflectance. For analysis here, we excluded data with $\chi^{2}<0.9$ for statistical analysis. This resulted in a loss of 28 DRS scans (10.4%) from the pain group and 69 scans (8.9%) from the no-pain group. After filtering the data based on this condition, multiple scans for shunt and scar sites for each patient remained. However, this process resulted in a reduction of contralateral scans available for statistical analysis (with three subjects from the pain group and two from no-pain group not having any contralateral site information).

### 2.4 Statistical Modeling

Optically derived values of vascular oxygen saturation (${\mathrm{SO}}_{2}$), total hemoglobin ([THb]), and wavelength-averaged reduced scattering ($<\mu_{s}>$) from each DRS scan (across all subjects and sites) were analyzed using a statistical model with repeated measures analysis of variance (ANOVA). The statistical modeling were performed using “lme4” package in R.^33^ To adjust the p-value of simultaneous inference, we conducted Tukey p-value adjustment in the *post hoc* analysis via “emmeans” package in R.^34^ These statistical techniques were selected to evaluate whether these optical variables were similar when compared across different sites from the same subject and influenced by the presence (or absence) of shuntodynia. The statistical model was selected to consider correlations present from repeated measurements at a given site and for different sites within the same subject.

Statistical comparisons were first made to identify if derived coefficients at different tissue sites within the same patient subset were separable (e.g., shunt versus contralateral comparisons for all patients with shuntodynia). Comparisons were not made to differentiate the pain versus no pain groups from each other but rather to compare site-level responses as a function of presence versus absence of shuntodynia. Results are presented from the modeled two-way repeated measures ANOVA.

## 3 Results

### 3.1 Optical Data

**Fig. 2:**
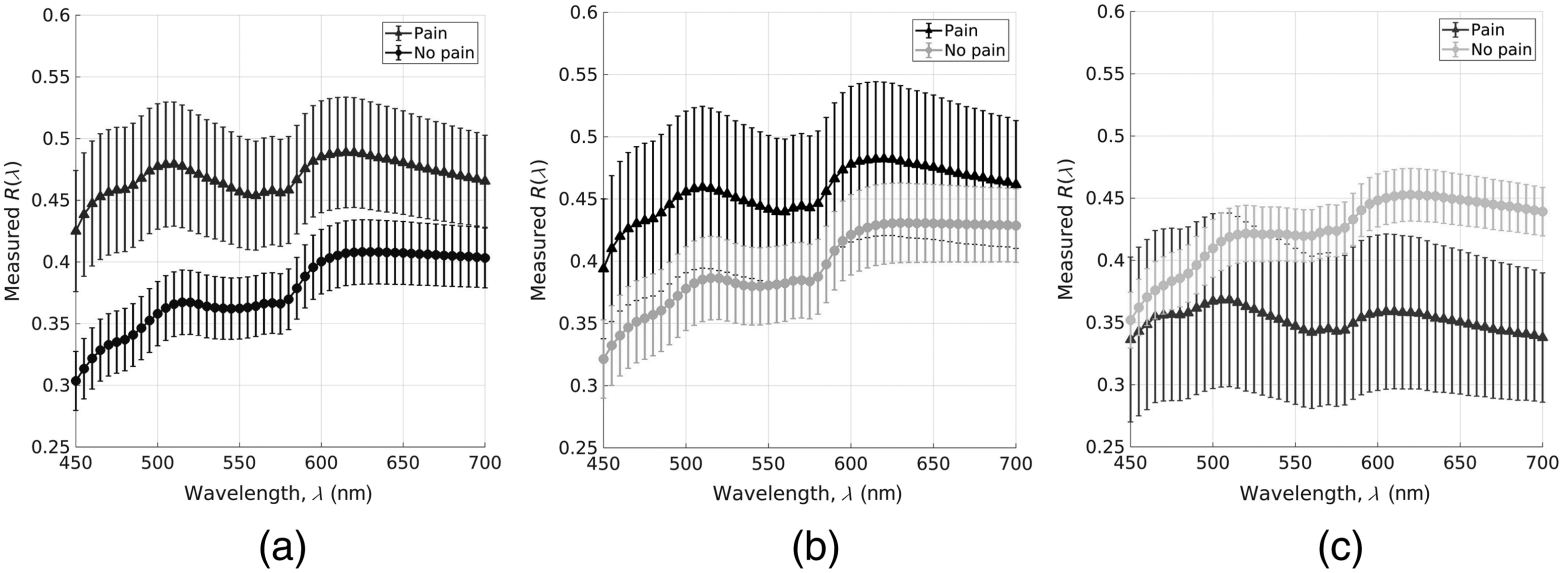
Averaged reflectance data across all patients in the pain (triangles) and no-pain (circles) groups obtained at the three different sites probed (a) DRS data at shunt sites, (b) DRS data at scar sites, and (c) DRS data on the contralateral side. Each figure shows mean DRS computed between patients in each group (symbols) and error bars are standard deviations.

Figure 2 shows the averaged DRS data collected from all subjects enrolled in the study based on the reported pain status and tissue sites [Figs. 2(a)–2(c) for sites around the shunt, the scar tissue, and a matched contralateral region, respectively]. The mean diffuse reflectance observed at both the shunt and scar sites, the shuntodynia group to have higher reflectance [Figs. 2(a) and 2(b)], which was not seen at the contralateral sites. Additionally, the variance across subjects was higher in the shuntodynia group in comparison to the no-pain group.

**Fig. 3:**
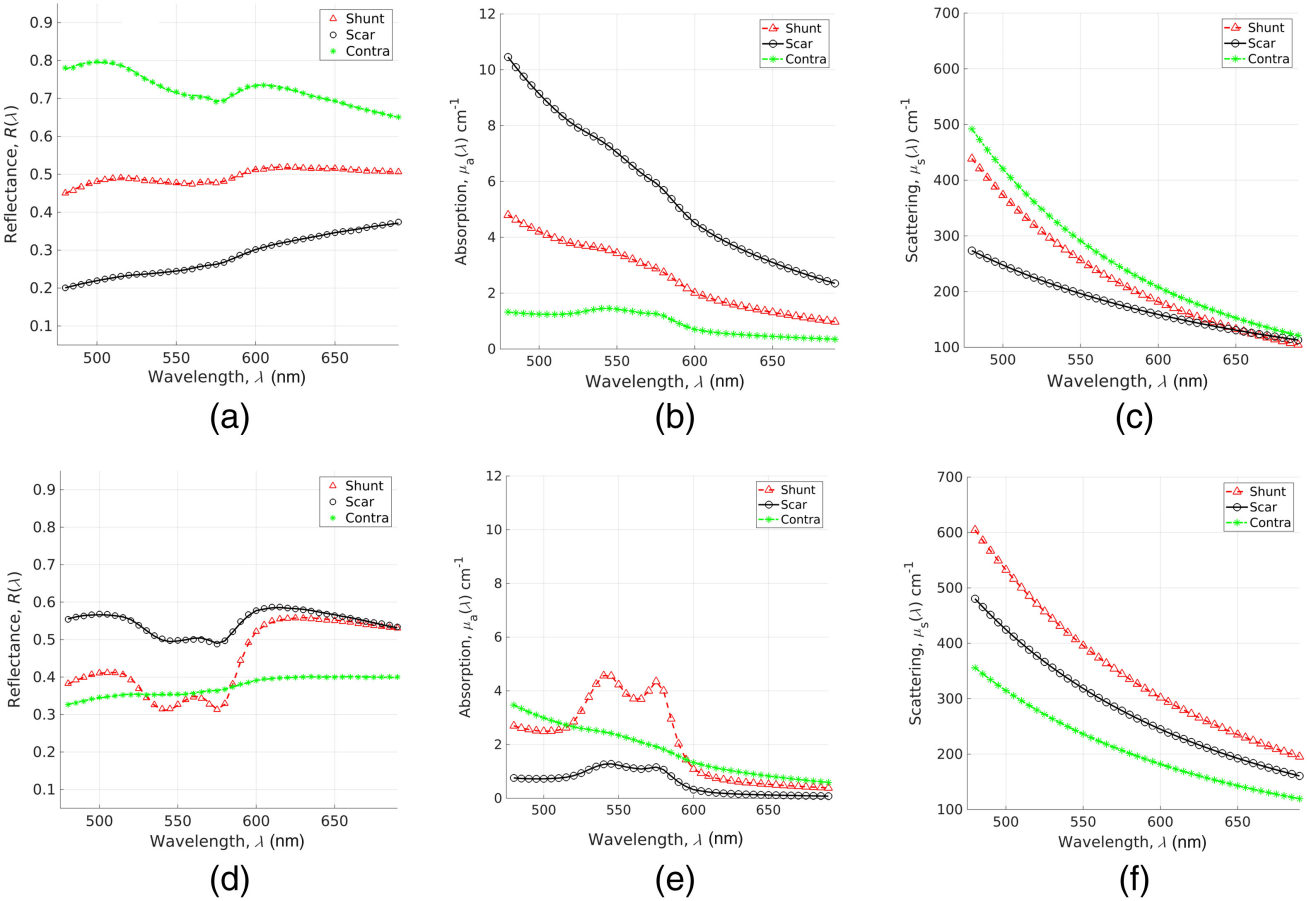
Measured (symbols) and fitted (lines) DRS data from two representative subjects [(a) with shuntodynia and (b) without shuntodynia]. (c), (e) Derived absorption and scattering coefficients for each DRS data fitted in (a). (d), (f) Data for data shown in (b). In each figure, shunt, scar, and contralateral data are shown using triangles, circles, and asterisks, respectively.

Each measured DRS spectrum was fitted and filtered for goodness of fits as in Eq. (1). Figure 3 shows the calibrated DRS measurements from two representative subjects [Figs. 3(a), 3(c), and 3(e) for a subject with shuntodynia and Figs. 3(b), 3(d), and 3(f) for a subject without], for each site when fitted by the inverse MC model. Symbols indicate measured values while lines indicate MC fits and all data shown here had $\chi^{2}$ values between 0.9 and 0.92. Figures 3(c) and 3(d) show the extracted absorption coefficients for the subject in pain and no-pain group, respectively [Figs. 3(e) and 3(f) show the derived scattering coefficients]. These optical coefficients were obtained from the inverse MC model as a result of fitting the data shown in Figs. 3(a) and 3(b), respectively, as discussed briefly above.^26^^,^^30^ As seen visually, measured data are well fitted by the inverse MC model and the extracted absorption spectra show well-defined characteristic features between 550 and 600 nm of oxygenated hemoglobin.

### 3.2 Hemodynamic Data

The absorption coefficients from inversely fitted DRS spectra were translated into the traditionally used optically derived functional endpoints of total hemoglobin ($\left[\mathrm{THb}]=[{\mathrm{HbO}}_{2}]+[\mathrm{dHb}\right]$) and vascular saturation percent (${\mathrm{SO}}_{2}=100\times [{\mathrm{HbO}}_{2}]/\mathrm{THb}]$), whereas the scattering coefficient was averaged across the spectrum to get the average scattering ($<\mu_{s}>$). Thus each DRS spectrum was reduced into three quantitative optical coefficients, which were analyzed statistically.

**Fig. 4:**
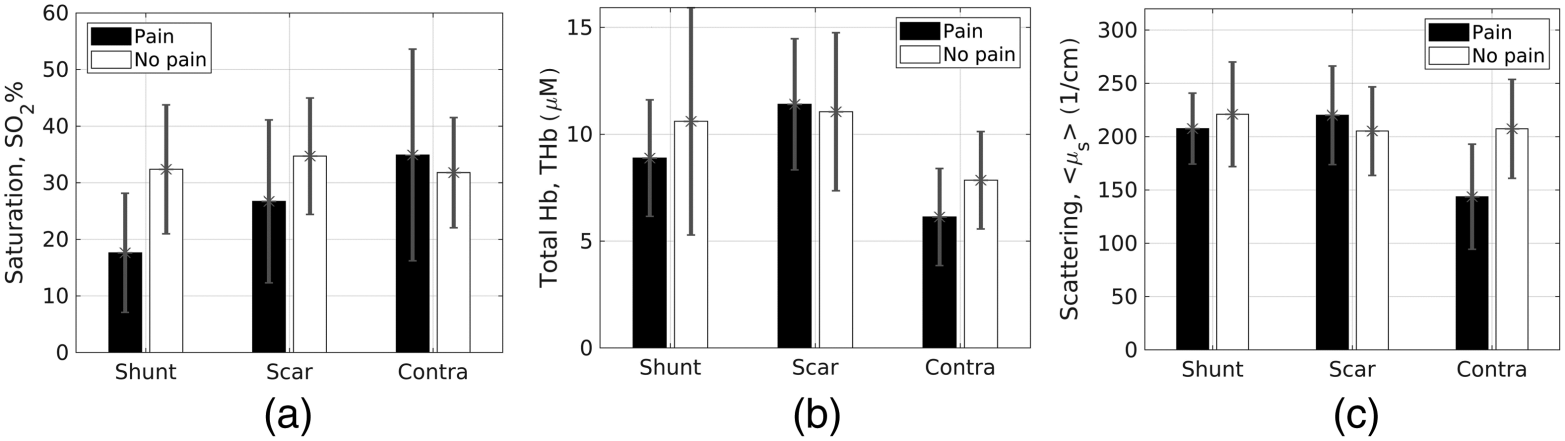
Comparison of mean optically derived hemodynamic and scattering coefficients across the three tissue sites for each patient group (black bars: with shuntodynia and white bars: without shuntodynia). Bars represent mean value while error bars depict standard deviations. (a) Data for the vascular oxygen saturation, (b) data for total hemoglobin concentration, and (c) data for the mean wavelength averaged reduced scattering.

Figure 4 shows a comparison of the three optically derived functional variables when averaged across each tissue site, for each patient group. The mean oxygen saturation and total hemoglobin values appeared to be similar between the shunt-scar sites for the patients that did not present with shuntodynia but those with shuntodynia had elevated total hemoglobin and oxygen saturation in the scar sites relative to the shunt sites.

Table 1 presents a summary of the statistical analysis used to model these data obtained and to facilitate site-wise comparisons within each patient group. Table 1 lists modeled means and standard errors in optical variables at each site, for both patient groups. Tukey-adjusted p-values are shown for comparisons of each optical variable between any two tissue sites as indicated by Table 1. Comparisons were made within the pain and no-pain groups with the intent of discovering if optically derived variables were different across tissue sites, within individual patients in each group. Statistical analysis was not performed to establish a difference between the pain and no-pain groups at the same site. The principal goal of our study was to identify if optically derived hemodynamic metrics could signal the presence (or absence) of shuntodynia, within individual subjects.

**Table 1:** Tukey-adjusted, pairwise comparisons of optical variables within patient groups with and without shuntodynia. Means and standard errors for the groups are shown as organized by site and optical variable. p-values are obtained from adjusted Tukey comparisons to estimate if mean differences in relevant variable-pairs across different sites were zero or not. NS indicates p-values above 0.05.

Patient group	Optical variable	Mean ± Std values at			p-values for site–site differences		
		Shunt	Scar	Contra–lateral	Shunt versus scar	Shunt versus contra	Scar versus contra
Shuntodynia	${\mathrm{SO}}_{2}$ (%)	17.9 ± 4.0	24.9 ± 4.3	35.4 ± 5.6	<0.05	<0.01	NS
	[THb] μM	8.7±1.6	11.2 ± 1.7	7.0 ± 2.1	0.05	NS	<0.05
	${<\mu }_{s}>{\mathrm{cm}}^{-1}$	213 ± 16	220 ± 18	143 ± 23	NS	<0.01	.01
No shuntodynia	${\mathrm{SO}}_{2}$ (%)	33.0 ± 2.4	35.4 ± 2.7	32.3 ± 3.0	NS	NS	NS
	[THb] μM	10.5 ± 1.0	11.0 ± 1.0	7.5 ± 1.2	NS	<0.01	<0.01
	$<\mu_{s}>{\mathrm{cm}}^{-1}$	222 ± 10	208 ± 11	205 ± 12	NS	NS	NS

Oxygen saturation proved to be the most useful metric in identifying pain within the group presenting with shuntodynia. For the pain group, oxygen saturation was significantly lower than both the scar and control sites. The extracted total hemoglobin coefficient also exhibited statistical significance within the shuntodynia group where this variable had elevated values at the scar site relative to the shunt sites. Finally, the control contralateral site showed significantly lowered scattering coefficient than all other sites for the patients experiencing pain and may be a statistical anomaly given the low number of contralateral scans available for this patient group.

Interestingly, in patient group without shuntodynia, oxygen saturation was not significantly different across any of the measured tissue sites. Likewise, the scattering coefficient also did not indicate any statistically significant differences between sites. However, total hemoglobin was elevated at both scar and shunt sites, which were located ipsilaterally on the patient’s heads as the implanted shunts. Though statistical comparisons were not performed between patient groups at the same site, Fig. 4 appears to indicate that the two patient groups could show differences at certain sites for some of these optical variables.

## 4 Discussion

Fiber-based DRS was used in a preliminary clinical study to objectively measure the hemodynamic status of skin tissue in pediatric patients that had implanted VP shunts as a part of their treatments for hydrocephalus. When recruited subjects self-reported feeling pain around the VP shunt, they were placed in the shuntodynia group while the others were placed into the no-shuntodynia group. The optically acquired measurements were translated into three functional endpoints of vascular oxygen saturation, the total hemoglobin concentration, and the wavelength averaged tissue scattering. These optical endpoints were then analyzed using a two-way ANOVA with a mixed-effects model to statistically model whether the presence of shuntodynia could be detected in hemodynamic signatures of individual subjects by comparing across different tissue sites, per-subject in each group. For each subject, DRS measurements were acquired from seven different tissue sites as described in Fig. 1. To the best of our knowledge, this work marks a first attempt to use quantitative DRS to extract optically derived functional metrics from the skin tissue surrounding implanted VP shunts for the objective detection of shuntodynia.

The acquired DRS spectra were fitted between 450 and 700 nm and our inverse fitting operated with the assumptions that the medium has homogeneous optical properties and that its absorption was a linear combination of four independent absorbers (oxygenated hemoglobin, deoxygenated-hemoglobin, eumelanin, and pheomelanin) whose extinction coefficients were known in the spectral window.^31^ It is important to note that the DRS data as obtained here can be analyzed by generic spectral classifiers (such as PCA or spectral decomposition) as well.^35^^,^^36^ However, the approach we use here seeks to quantitatively establish hemodynamic parameters using a physics-based light transport model and then explore if these hemodynamic markers could statistically be linked to the presence (or absence) of pain. As mentioned previously, our DRS data were obtained experimentally to be localized to the superficial layers of scalp tissue, rather than deeper tissue, such as skull or the brain using optical fibers separated by 600  μm. Thus our extracted optical coefficients would represent scalp tissue properties more than the skull.

However, our experimental protocol only consisted of obtaining repeat scans at each tissue site for each subject and from these data, we observed that coefficient of variance in the DRS data across the spectrum of interest for repeated scans at a site was clearly lower than the coefficient of variance calculated between sites, which itself was comparable to the variance between subjects. This gives confidence that we had consistent data acquisition in our experimental protocol but does not establish reproducibility. The question of reproducibility would require a redesign of the experimental protocol and will be investigated in the future.

Selection of the random effects model used in the statistical analysis of hemodynamic endpoints incorporated any potential correlations in measurements—both within a site and across different sites in an individual. Therefore, both interpatient and intrapatient variabilities are fully considered in the statistical methods used to model extracted hemodynamic data and thus our analyses accounts for uncertainties and correlations present in the data within and between sites. The analysis shows that only for group of subjects that reported pain the oxygen saturations at the shunt sites was significantly lower relative to the scar or contralateral sites. Patients that did not report pain did not exhibit statistically significant differences in vascular oxygenation (see Table 1). For the total hemoglobin variable, we observed that in subjects that did not report shuntodynia, THb was significantly higher at the shunt and the scar sites than it was for the contralateral side. But for patients reporting shuntodynia, THb was only significantly higher at the scar sites than it was at the contralateral sites—the shunt site had lowered THb for this group, further lending credence to the idea of the vascular supply or function being adversely impacted for this group. In the no-shuntodynia group, there was greater variability across THb estimated at the shunt and scar sites. Overall, there were no discernable differences in the mean scattering for patients with versus without shuntodynia between shunt and scar sites. But we did observe a statistically significant difference for scattering measurements between the contralateral versus scar or shunt sites, in the pain group. Given the low number of scans available for contralateral measurements, this conclusion remains to be validated by future studies.

As described above, we relied on the goodness of fit metric [Eq. (1)] to reject poor-quality fits of measured data. This was done to only consider hemodynamic variables that were extracted from measurements that could be well fitted by inverse MC predictions. We hypothesized that these low-quality fits could be from experimental problems during collection from subjects—particularly with difficulties with keeping the optical probe in firm physical contact with the skin tissue for DRS acquisition. There could be information potentially retrieved from some of these discarded DRS scans here, perhaps using other heuristic approaches to assess hemodynamic variables but we elected to use a more conservative approach given the preliminary nature of the work.

Of the three optical variables derived from measured DRS data, oxygen saturation presented as the most promising metric for identifying shuntodynia. Not only was there a distinction between the pain and no pain groups for oxygen saturation, but pain patients exhibited a lower oxygen saturation around their shunt sites. We focused on using vascular signals detected superficially from scalp and skull tissue using DRS. This was motivated by a hypothesis that the experience of pain felt by patients emerged from the skin-sensory system below which VP shunts were implanted. In shuntodynia patients, the sensation of pain appears localized in the vicinity of the shunt, which we suspect is due to motion of the shunt under the skin, along with local pressure changes experienced by fluid flowing through the shunt. Given the sample size of our study along with the techniques used, we cannot conclude that it was the reduction of vascular oxygenation that causes shuntodynia, though previous reports using optical spectroscopy have also observed that subjects with reduced vascular oxygenation reported pain and have suggested that it could be caused by tissue hypoxia.^37^ Another study has reported that patients suffering from complex regional pain syndrome type 1 have reduced oxygen vascular saturation in their affected hand when compared to their unaffected contralateral hand.^17^ This finding could also be supported by reported assertions of oxygen saturation being a potential biomarker of pain, whether directly related to increased sympathetic activity versus hypovolemia.^38^

When the results of our study here are taken together with these previously published reports, it appears likely that the shunt placement together with its motion could be causing tissue inflammation and hypoxia in subjects with shuntodynia. Future explorations will consider use of other well-established noncontact optical imaging methods (such as spatial frequency domain methods) to measure vascular functions. Since shuntodynia was self-reported by the patient, our use of DRS here to measure pain was exploratory. However, we have succeeded in our intent, which was to first establish whether DRS was sensitive to presence of self-reported pain in VP shunt patients. In the future, we will consider if such methods could be adapted to explore fundamental pathways from where the painful stimuli emerge.

## 5 Conclusion

Here we present preliminary findings for the first time that suggests there were differences in optically estimated hemodynamic signatures in scalp tissues of pediatric hydrocephalus patients that experienced shuntodynia post-VP shunt implantations. Our results showed that oxygen saturation held the greatest statistical value for identifying the presence of pain called shuntodynia. Though the use of DRS was direct and noninvasive, the fiber-based system required contact with skin tissue. As a practical issue in this study, the clinical condition naturally presented impediments to establishing good contact between the probe and skin tissues in subjects and took significant effort and costs in terms of clinical time and patient management for appropriate optical data collection. Thus having fully noncontact optical methods—such as spatial frequency domain imaging or hyperspectral imaging—for sensing tissue hemodynamics could vastly improve data collection ability, especially when working with patients experiencing pain. Additionally, it will also be valuable to integrate optical tissue perfusion measures, such as diffusion correlation spectroscopy, to build a more complete picture of subcutaneous hemodynamics in shuntodynia.

## Data Availability

Programs written to facilitate the process of obtaining scans and also inverting them via the inverse Monte-Carlo model were developed in-house and written in MATLAB. Code used to analyze and model the hemodynamic variables with the two-repeated measures ANOVA were written in R.
